# Neurofibromatosis 1 French national guidelines based on an extensive literature review since 1966

**DOI:** 10.1186/s13023-020-1310-3

**Published:** 2020-02-03

**Authors:** Christina Bergqvist, Amandine Servy, Laurence Valeyrie-Allanore, Salah Ferkal, Patrick Combemale, Pierre Wolkenstein, NF France Network, Henri Adamski, Clarisse Baumann-Morel, Christine Bellanné, Eric Bieth, Pascal Bousquet, Christian Brandt, Xavier Balguerie, Sébastien Barbarot, Pierre Castelnau, Yves Chaix, Jacqueline Chevrant-Breton, Evelyne Collet, Jean-François Cuny, Pascal Chastagner, Marie-Lorraine Chandeclerc, Emmanuel Cheuret, Pascal Cintas, Helene Dollfus, Christian Derancourt, Valérie Drouin-Garraud, Michel d’Incan, Hélène De Leersnyder, Olivier Dereure, Diane Doumar, Nicolas Fabre, Vincenza Ferraro, Christine Francannet, Laurence Faivre, Florence Fellmann, Nathalie Feugier Dominique Gaillard, Alice Goldenberg, Lucie Guyant-Marechal, Bernard Guillot, Jean-Sebastien Guillamo, Smaïl Hadj-Rabia, Dominique Hamel-Teillac, Isabelle Kemlin, Jean-Philippe Lacour, Veronique Laithier, Nathalie Lesavre, Stanislas Lyonnet, Kim Maincent, Sophie Maradeix, Juliette Mazereeuw-Hautier, Laurent Machet, Eva Mansat, Nicolas Meyer, Monique Mozelle, Jean Christophe Moreno Celine Moret, Eric Puzenat, Béatrice Parfait, Stéphane Pinson, Eric Pasmant, Diana Rodriguez, Jean-François Stalder, Emilie Sbidian, Elisabeth Schweitzer, Claire Thalamas, Christel Thauvin, Dominique Vidaud, Michel Vidaud, Alain Verloes, Ouidad Zehou, Jacques Zeller

**Affiliations:** 1 Faculty of medicine, Université Paris-Est Creteil (UPEC), F-94010 Créteil Cedex, France; 2 Assistance Publique-Hôpital Paris (AP-HP), Hôpital Henri-Mondor, Service de Dermatologie, F-94010 Créteil, France; 3 INSERM, Centre d’Investigation Clinique 006, Referral Center of Neurofibromatosis, Assistance Publique-Hôpital Paris (AP-HP), Hôpital Henri-Mondor, F-94010 Créteil, France; 4 Rhône-Alpes Auvergne Competence Center for the treatment of Neurofibromatosis type 1, Léon Bérard Comprehensive Cancer Center, Hôpitaux Universitaires de Lyon, Université de Lyon, F-69008 Lyon, France

**Keywords:** Neurofibromatosis type 1, Guidelines, Management, Multidisciplinary, Diagnosis, Genetic counseling, Dermatology, Orthopedics, Neurology, Oncology, Malignant peripheral nerve sheath tumors, Optic glioma, Quality of life

## Abstract

Neurofibromatosis type 1 is a relatively common genetic disease, with a prevalence ranging between 1/3000 and 1/6000 people worldwide. The disease affects multiple systems with cutaneous, neurologic, and orthopedic as major manifestations which lead to significant morbidity or mortality. Indeed, NF1 patients are at an increased risk of malignancy and have a life expectancy about 10–15 years shorter than the general population. The mainstay of management of NF1 is a patient-centered longitudinal care with age-specific monitoring of clinical manifestations, aiming at the early recognition and symptomatic treatment of complications as they occur. Protocole national de diagnostic et de soins (PNDS) are mandatory French clinical practice guidelines for rare diseases required by the French national plan for rare diseases. Their purpose is to provide health care professionals with guidance regarding the optimal diagnostic and therapeutic management of patients affected with a rare disease; and thus, harmonizing their management nationwide. PNDS are usually developed through a critical literature review and a multidisciplinary expert consensus. The purpose of this article is to present the French guidelines on NF1, making them even more available to the international medical community. We further dwelled on the emerging new evidence that might have therapeutic potential or a strong impact on NF1 management in the coming feature. Given the complexity of the disease, the management of children and adults with NF1 entails the full complement healthcare providers and communication among the various specialties.

## Background

Neurofibromatosis type 1 (NF1) is one of the most common inherited disorder. Most epidemiological studies have reported a prevalence ranging between 1/3000 and 1/6000 [1–4], and birth incidence estimates between 1/2558 and 1/3333 [1, 2, 5–7]. Recent evidence revealed that NF1 is a much more common disorder than previously thought, with a birth incidence of 1:2000 [7] and a prevalence of 1/4000 [4]. NF1 is a multisystem genetic disease that is principally associated with cutaneous, neurologic, and orthopedic manifestations; some of which are progressive and lead to significant morbidity or mortality. NF1 patients are at an increased risk of malignancy and have a life expectancy about 10–15 years shorter than the general population [8–14]. A total population study in Finland demonstrated that NF1 reduces the life expectancy of women considerably more than that of men; with a life span shortened by 16.5 years in men and by 26.1 years in women with NF1 [7].

The mainstay of management of NF1 is a patient-centered longitudinal care with age-specific monitoring of clinical manifestations, aiming at the early recognition and symptomatic treatment of complications as they occur. Active engagement and an active partnership among multiple health care providers, concerned lay groups and patient experts is the cornerstone of management of this rare disease.

In 2005, the French National Authority for Health (Haute Autorité de Santé) called for the establishment of clinical practice guidelines for rare diseases (protocole national de diagnostic et de soins; PNDS). The purpose of a PNDS is to provide health care professionals with guidance regarding the optimal diagnostic and therapeutic management of patients affected with a rare disease; and thus, harmonizing their management nationwide. PNDS are usually developed through a critical literature review and a multidisciplinary expert consensus (www.has-sante.fr).

**Fig. 1:**
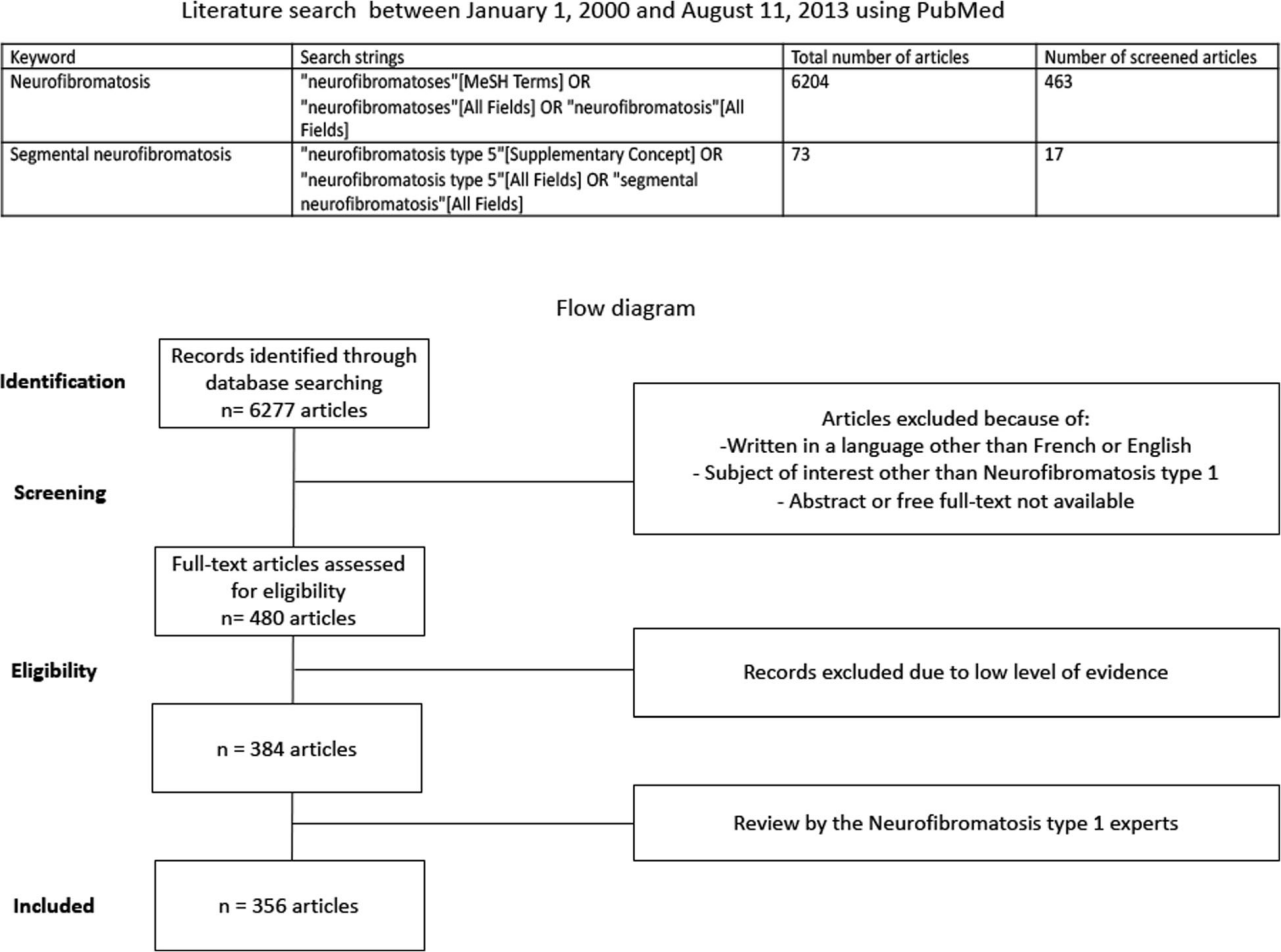
Literature search strategy and flow diagram

The PNDS on NF1 was written by the French expert group on NF1, NF-France Network (réseau NF-France) and published in December 2016. The previous recommendations were based on a literature review extending from 1966 to 1999 [15, 16]. The currently updated recommendations were based on an extensive review of the literature that spans between January 1, 2000 and August 11, 2013. Given the ever-evolving nature of research, we also performed a judicious and critical literature review from August 2013 to November 2018 to shed the light on the emerging new evidence that might have therapeutic potential or a strong impact on NF1 management. These have been incorporated under the “emerging evidence” sections after each paragraph. These are likely to be included in the next updated version of the PNDS, as these guidelines are continuously updated based on the constantly evolving scientific evidence. The current published PNDS used PubMed as a search engine and the keywords “neurofibromatosis“ and “segmental neurofibromatosis“. Search strings incorporated both Medical Subject Headings (MeSH) and free text key words (Fig. 1). Only relevant articles published in French or English, and for which an abstract or the full text was freely available were retained (*n* = 6277). Titles and/or abstracts of studies were screened independently by two review authors to identify studies that met the inclusion criteria. After screening titles, abstracts, full-text articles; and then looking in particular at their level of evidence, 384 articles were suitable for inclusion. Included articles underwent data extraction and were graded according to the Haute Autorité de Santé Evidence-based Medicine criteria (https://www.has-sante.fr/upload/docs/application/pdf/2013-06/etat_des_lieux_niveau_preuve_gradation.pdf). Two review authors extracted data independently, discrepancies were identified and resolved through discussion or with a third author when deemed necessary. Included articles had the following data extracted (when applicable): study characteristics, including design, setting/data source and study period; participant characteristics, such as sample size, mean age, sex, mean follow-up; baseline characteristics, treatment and quality of life questionnaires.

A first draft was written and then submitted for review to various NF1 experts, who in turn, based on their own professional expertise, added missing references if any. The study flow diagram is shown in Fig. 1. The full text along with the list of the multidisciplinary working group participants can be found on the Haute Autorité de Santé website (https://www.has-sante.fr/portail/jcms/c_2734080/fr/neurofibromatose-de-type-1).

The purpose of this article is to present the French guidelines on NF1, making them even more available to the international medical community. This article focuses mainly on the major management strategies of the PNDS, trying to be as exhaustive as possible.

## Diagnosis

### Clinical diagnosis

In general, NF1 can be diagnosed by physical examination and by evaluation of the patient’s family history. NF1 diagnosis relies primarily on the basis of the National Institutes of Health (NIH) diagnostic criteria [17]. 97% of NF1 patients meet the NIH criteria by the age of 8 years, and all do so by the age of 20 years [18]. These criteria usually appear in the following predictable order: café-au-lait macules, axillary freckling, Lisch nodules, and neurofibromas. The characteristic osseous lesions usually appear within the first year of life, and the mean age at diagnosis of optic gliomas ranges from 3 to 6 years [18–20].

#### Emerging evidence

Revising these diagnostic criteria is currently a hot topic in the NF1 community, since the NIH diagnostic criteria have been proven to be inadequate in establishing a diagnosis at an early age. Only 50% of children with sporadic NF1 younger than 2 years fulfil only a single NIH criterion, often leading to a delay in the diagnosis [18, 21]. Some authorities have suggested to include other clinical signs in addition to the NIH criteria for the diagnosis of NF1 including cutaneous signs and extra-cutaneous signs (large head circumference, unidentified bright objects among others) [22]. Juvenile xanthogranulomas (JXG) and nevus anemicus are present in most NF1 children aged younger than 2 years and were found in 80% of patients with insufficient criteria for diagnosis [23]. Therefore, JXG and nevus anemicus are helpful criteria in improving the early diagnosis of NF1 in young children and infants. Moreover, multiple café-au-lait macules (CALMs) can be a presenting feature of other syndromes. Legius syndrome is an autosomal dominant disorder caused by the loss-of-function *SPRED1* mutations. It is characterized by multiple CALMs with or without freckling and absence of neurofibromas, Lisch nodules, and lack of high prevalence of malignancies [24, 25]. In a study of 71 patients younger than 20 years of age with six or more CALMs and no other criterion, 66.2% were discovered to have NF1, 8.5% had Legius syndrome and 25.3% harbored no disease causing variant [26]. Genetic testing can therefore be helpful in confirming the diagnosis of NF1 for children with multiple CALMs and axillary freckling who do not meet other diagnostic criteria.

### Genetic testing

So far, the diagnosis of NF1 relies primarily on clinical grounds and genetic testing is not needed when the diagnosis has already been established. Genetic testing can be particularly helpful for patients who present with an unusual phenotype or an incomplete clinical picture [27]. It can also be of great advantage in children presenting with multiple CALMs as the sole clinical feature with no family history of NF1, to be able to differentiate the diagnosis of NF1 from other syndromes such as Legius syndrome and Noonan syndrome [26]. Genetic testing also helps in delivering a suitable genetic counseling for parents regarding any future planned pregnancy. A vast number of different pathogenic *NF1* mutations have been described [28–32] and molecular testing with high sensitivity is currently clinically available [28, 30–33]. It is noteworthy, however, that a specific NF1 mutation does not predict the severity or complications of the disease. Indeed, no straightforward genotype–phenotype correlations have been identified for patients with intragenic NF1 mutations [34–36] with a few reported exceptions [37–39].

In 5–10% of patients, NF1 results from microdeletions that encompass the entire NF1 gene and a variable number of flanking genes [40–42]. These large NF1 locus deletions have been associated with a more severe phenotype including developing neurofibromas at an earlier age, having a lower mean IQ, abnormal facial features, and an elevated risk for malignant peripheral nerve sheath tumors (MPNST) [43–45].

#### Emerging evidence

Many NF1 experts believe that the diagnosis should include molecular testing as it leads to early recognition of NF1 in children and allows for appropriate surveillance. While traditional molecular analysis methods (using cDNA and/or DNA Sanger sequencing and copy number alteration studies) were able to identify around 95% of NF1 gene mutation s[28, 30–33], a new targeted next-generation sequencing of *NF1* and *SPRED1* using a multiplex PCR approach was recently introduced with a sensitivity up to 98.5% [46].

### Announcing the diagnosis

Announcing the diagnosis of a genetic disorder such as NF1 is a critical event in the lives of both the child and the parents. It can often be distressing, eliciting strong emotions such as the anxiety of an unknown disease in non-familial forms, guilt in familial forms; and for all, apprehension of the prognosis and potential complications. Disclosing the diagnosis should be done in the setting of a well-planned, dedicated, face-to-face consultation which requires expertise and unlimited time. It should be tailored to the family history, whether familial or sporadic. The parents should be referred to a specialist in genetic counseling and a well-trained psychologist for a more comprehensive discussion of clinical outcomes, social and psychological support and future reproductive options.

Depending on the age of the patient, the natural history, clinical picture, variability, prognosis, personalized treatment, complications and the warning signs that should prompt rapid medical attention must be reviewed with the child and parents. They should also be provided with the most recent scientific advances and the latest therapeutic and supportive care options, including their efficacy and limitations; as well as available validated NF1 resources such as books, pamphlets, reliable website addresses and support groups. Finally, they should be informed about the available neurofibromatosis foundations, centers and clinics for further guidance and multidisciplinary care.

Another essential element in the announcement is to address the possibility of extension of the disease to other family members.

### Genetic counseling

NF1 is a fully penetrant autosomal dominant genetic disorder (no skipped generations or asymptomatic carriers) [3]. A parent with the disease has a 50% chance of having a child with NF1 [27]. A detailed family history should be obtained once the diagnosis of NF1 is made in a child. Genetic counseling should be offered to NF1 patients and all first-degree relatives who desire it. Once the causative NF1 mutation has been identified in the parent, prenatal and pre-implantation genetic testing can be offered. However, since NF1 has a variable expressivity, it is usually not possible to predict the severity of the disease.

- NF1 diagnosis relies primarily on clinical grounds using the NIH criteria.
- Genetic testing is useful in patients who do not meet these diagnostic criteria.
- Disclosing the diagnosis should be done by an expert in the setting of a dedicated consultation.
- The natural history, prognosis, personalized treatment, complications of the disease and the warning signs that should prompt rapid medical attention must be reviewed with the child and parents.
- Genetic counseling should be offered to NF1 patients and all first-degree relatives who desire it.

## Principal NF1 manifestations and their management

### Dermatological manifestation

#### Benign manifestations

**Café-au-lait macules** are usually present at birth and occur in > 90% of patients [18]. They are large, generally oval, well-defined hyperpigmented macules.

**Skinfold freckling** are found in > 80% of NF1 individuals [47, 48]. They can appear in any area where skinfolds are in apposition, including the axilla, intertriginous area, base of the neck, upper eyelid and under the breasts in women [49].

CALMs and skinfold freckling have no malignant potential and the family should be reassured that these have no functional significance.

**Neurofibromas (NF)** are benign peripheral nerve sheath tumors and are the cardinal feature of NF1.

There are four major types of NF:

Cutaneous (or dermal) NFs are soft flesh-colored or purplish nodules that may become pedunculated as they grow. They usually develop in late adolescence and are found in the vast majority (> 95%) of patients with NF1 [50, 51]. They vary in number from a few lesions to thousands [50]. Although these skin tumors are benign and have no risk of malignant transformation, they can cause significant discomfort and cosmetic disfigurement.

Management is only recommended for cases with severe clinical manifestations and/or esthetic discomfort with secondary psychological repercussions. Treatment options depend on the number of lesions and their location. First line treatments include surgical excision and/or CO2 laser ablation. The latter can be particularly helpful for small lesions on the face and neck [48, 52]. Second line treatments include radiofrequency ablation and electrodessication. Electrodessication is a useful tool as it enables the treatment of hundreds of neurofibromas in a single operation under general anesthesia, with low complication rates and high levels of clinical and patient-reported outcomes [53, 54].

Subcutaneous neurofibromas (or peripheral nodular NFs) are firm discrete palpable lesions. They affect at least 20% of NF1 patients and usually develop during adolescence [50]. They appear as firm rubbery nodules bulging under the skin. These lesions develop along the path of nerve trunks. They may be tender to touch, and can cause tingling along the affected nerve, or even neurological deficits.

Internal (nodular) NFs are neurofibromas that cannot be appreciated by physical examination [55]. These are associated with a “high-risk phenotype“, since MPNSTs can develop from internal neurofibromas, warranting closer clinical monitoring and serial MRI examinations for changes in the appearance or growth of internal tumors to allow earlier diagnosis and more effective treatment of MPNSTs in these high-risk patients (See below) [56, 57]. A rarer form, with a deeper location (mostly paraspinal) exists and is associated with a poorer prognosi s[58]. Surgical intervention should be undertaken on the grounds of severe pain, progressive neurological symptoms and risk of permanent deficit [48].

Plexiform neurofibromas are congenital and are present in 20 to 26% of individuals with NF1 [59]. These lesions present as a subtle enlargement of soft tissue with a “wrinkled” texture or a patch of hyperpigmentation with or without hypertrichosis. Considerable increase in size subsequently follows during the first decade of life and adolescence [60]. Internal plexiform neurofibromas can be found in up to 50% of NF1 patients when using whole-body imaging [57, 61]. These tumors can invade surrounding structures, including muscle and bone and can lead to significant pain and bone destruction [62]. Plexiform neurofibroma of the face may be potentially devastating and associated with underlying hemi-hypertrophy and sphenoid dysplasia [63]. Plexiform neurofibromas may remain asymptomatic; however, they can also cause significant morbidity such neurological deficit, disfigurement and pain [64]. These tumors have a lifetime risk of malignant transformation into MPNST [60]. Management of these lesions is complex and is particularly recommended in case of morbidity and/or esthetic discomfort with psychological impact. Radiotherapy is contraindicated in benign tumors due to the risk of secondary malignant degeneration [48]. Surgical excision is the first line treatment; however, expert advice should be thought from experienced surgeons as the excision is often very challenging due to the impingement of the tumor on contiguous nerves and structures and its characteristic extensive vascularity that can result in life-threatening hemorrhage [65]. Experts recommend the early excision of plexiform NFs with the potential to cause morbidity, as to limit their functional and esthetic impact, including preventing the risk of malignant transformation. Early excision as the lesions are smaller offers the advantages of a lower risk with a safer surgical approach [65, 66].

Atypical neurofibromas (ANF) are classified histologically as lesions that have increased variable cellularity, more cytological atypia, and more pronounced fascicular growth patterns as compared to the more typical neurofibroma; but lack the widespread monotonous cytological atypia, the fascicular growth mitotic activity and the necrosis seen in MPNST [67, 68]. A deletion at 9p21.3, which includes genes CDKN2A/2B, was identified in 15/16 (94%) ANF and in 16/23 (70%) high-grade MPNST but not in plexiform neurofibromas; supporting the hypothesis that ANF are premalignant tumors, with the CDKN2A/B deletion as the first step in the progression toward MPNST [69]. This makes early detection and management of ANF a possible strategy to prevent MPNST [70].

##### Emerging evidence

The past years have seen advancements in the medical treatment of plexiform neurofibromas. Several treatments have been studied in clinical trials thanks to the advances in volumetric magnetic resonance imaging (MRI) along with whole body MRI which permit an accurate assessment of tumor response to a new therapy [71, 72]. The farnesyl transferase inhibitor tipifarnib [73, 74], the mammalian target of rapamycin inhibitor sirolimus [75, 76], and the fibroblast inhibitor pirfenidone [77] did not provide enough benefit to support their use. Imatinib and pegylated interferon have both proven to cause a reduction in size of plexiform neurofibromas in a limited number of patients [78–80]. A phase 1 study with selumetinib, an oral selective mitogen-activated protein kinase inhibitor has shown promising results whereby NF1 children with inoperable plexiform neurofibromas benefited from long-term dose-adjusted administration of selumetinib; a treatment which is generally well tolerated [81].

**Juvenile xanthogranulomas (JXG)** are benign yellowish to brownish papules. They are usually present in children younger than 2 years with NF1 and generally spontaneously disappear before the age of 5 years [82]. An association between NF1, multiple JXG and juvenile myelomonocytic leukemia (JMML) has been reported in few studies [83–85]; however, JMML is extremely rare and this association remains controversial [85]. Physicians should be aware of the presenting signs and symptoms of JMML and clinical examination must be thorough and directed.

**Glomus tumors** have also been associated with NF1, they are usually multiple and recurring [86, 87] and should be differentiated from symptomatic subcutaneous neurofibroma s[88, 89]. They cause localized tenderness, severe paroxysmal pain, and sensitivity to cold [89]. Surgical excision should be considered if the lesion is painful.

**Nevus anemicus** are congenital hypopigmented, confluent, and mottled macules mostly found on the anterior chest wall and are found in up to 50% of patients [90].

#### Malignant manifestations

**Malignant peripheral nerve sheath tumors (MPNST)**

MPNST are a subtype of sarcoma. NF1 patients have a cumulative lifetime risk of developing MPNST of 8–16% and occurs mostly at ages 20–35 years [12, 88, 91–93]. Most, if not all, MPNSTs in patients with NF1 appear to develop from preexisting plexiform neurofibromas or non-dermal neurofibromas which have undergone malignant transformation [88, 94, 95]. Seventy percent of these tumors are of high-grade that can metastasize widely and entail a poor prognosis [91]. The median survival is 18 months and the 5 years survival is 21% [88]. Symptoms most suggestive of MPNST are persistent, substantial or difficult to control pain, new neurological deficit, a rapid increase in the size of an existing plexiform neurofibroma or alteration in its consistency from soft to hard [91]. Risk factors for MPNST include a large internal neurofibroma burden or numerous subcutaneous neurofibromas, atypical neurofibromas, and neurofibromatous neuropathy [43, 44, 91, 96–98]. Factors associated with a poor MPNST prognosis are shown in Table 1.

**Table 1:** Factors associated with a poor MPNST prognosis

Factors associated with a poor MPNST prognosis	
Clinical and radiological	Site: axial/trunk [99–101]
	More than one primary tumor [102]
	Larger tumor size [99, 101–103]
Histological	High histological grade [101, 102]
Genotypic	Telomerase activity and overexpression of TERT[104]
	Genomic changes in chromosomes 10, 16, and X [105]

*TERT* Telomerase reverse transcriptase

MPNSTs should be suspected in a firm and rapidly growing neurofibromas that cause persistent or nocturnal pain, or a neurological deficit. MRI helps define the size and location of the lesion but cannot easily differentiate between benign and malignant tumors. The most sensitive and specific noninvasive indicator of malignant potential is [18F]2-fluoro-2-dexoy-D-glucose positron emission tomography computed tomography (FDG PET CT) using visual assessment and semiquantitative assessments with a cut-off SUV [106–109]. Biopsy should be MRI-guided or FDG PET CT-guided as the heterogeneous nature of some MPNST makes it likely for blind biopsy to miss the area of malignant change in a tumor with mixed features [91].

Once the diagnosis of MPNST is suspected, patients should be evaluated and managed by a multidisciplinary team including surgeons, radiologists, pathologists, oncologists, neurologists and radiation oncologists to efficiently apply a strategy for biopsy and treatment. The best treatment option is the complete surgical resection of the MPNST with tumor-free margins (3 cm if possible) [110]. Radiotherapy provides local control and could delay the onset of recurrence but doesn’t have an impact on the long-term survival [91]. Palliative radiotherapy can be used in patients with an incomplete resection or unresectable tumor. Therapeutic agents used for the treatment of MPNST include those usually used to treat sarcomas such as doxorubicin, trabectedin, ifosfamide, dacarbazine and pazopanib. Neoadjuvant chemotherapy with ifosfamide and an anthracycline such as doxorubicin can be administered to downstage tumors and facilitate surgical removal; however, this practice hasn’t been widely adopted [91, 111, 112]. Adjuvant chemotherapy using the same combination also remains controversial [113]. Single-agent anthracycline is often used as front-line therapy for palliative care in patients with metastatic disease [91, 112].

Follow up requires both clinical examination and imaging, the frequency of which is determined by the tumor site and its histological grade. Expert opinion recommends following patients every 3 months for 3 years, then every 6 months of 2 years then annually.

##### Emerging evidence

A wide variety of therapies is currently being investigated in clinical trials (including targeted therapies and immunotherapy). PET CT was shown to be a useful tool for the evaluation of treatment response and for the differentiation of tumor recurrence from the secondary effects arising from radiotherapy [114]. Furthermore, recent studies have shown that the specificity of detecting MPNST using FDG PET could be significantly increased by using a tumor-to-liver uptake ratio [115, 116]..

- The skin of NF1 patients harbors mostly benign lesions including CALMS, skinfold freckling, neurofibromas, JXG, glomus tumors and nevus anemicus.
- Neurofibromas are divided into different types: cutaneous (dermal), subcutaneous (peripheral nodular), internal (nodular) NFs, and plexiform NFs.
- Cutaneous (dermal) NF are found in the vast majority of patients. Management is only recommended for cases with severe clinical manifestations. First line treatments include surgical excision and/or CO2 laser ablation. Electrodessication is useful for the treatment of hundreds of neurofibromas at once.
- Subcutaneous (peripheral nodular) neurofibromas are firm rubbery nodules bulging under the skin. They are found in 20% of NF1 patients and usually develop during adolescence. They may be tender to touch, and can cause tingling along the affected nerve, or even neurological deficits.
- Internal (nodular) NFs are neurofibromas that cannot be appreciated by physical examination. They are associated with a “high-risk phenotype“, since MPNSTs can develop from internal neurofibromas, warranting closer monitoring and serial MRI examinations. Surgical intervention should be undertaken on the grounds of severe pain, esthetic distress with secondary psychological repercussions, progressive neurological symptoms and risk of permanent deficit.
- Plexiform NFs can invade surrounding structures and have the potential to degenerate into MPNST. Surgical excision is the first line treatment. Experts recommend the early excision of plexiform NFs as to limit their functional and esthetic impact. Early excision offers the advantages of a lower risk with a safer surgical approach.
- Persistent, substantial or difficult to control pain of an existing plexiform neurofibroma, new neurological deficit, a rapid increase in the size or an alteration in its consistency from soft to hard are signs of possible malignant transformation into a MPNST. It is crucial to educate patients how to recognize important symptoms and to seek specialist advice promptly.
- MPNST are a subtype of sarcoma. The majority of these tumors are high-grade and entail a poor prognosis. The most sensitive and specific imaging is FDG PET CT. Biopsy should be MRI-guided or FDG PET CT-guided. The best treatment option is the complete surgical resection of the MPNST with tumor-free margins (3 cm if possible)

### Ophthalmological manifestations

**Lisch nodules** are pigmented iris hamartomas that start developing around the age of 3 years and are found in 100% of patients by the age of 30 years [18, 117, 118]. They are asymptomatic 1–2 mm yellow brown dome shaped papules of the iris; and are best visualized using careful slit-lamp examination of the non-dilated iris.

The choroid is one of the most commonly affected structures by NF1. **Choroidal abnormalities** are visualized using near-infrared reflectance and appear as bright patchy nodules [119]. They have been recognized as being a highly specific finding for NF1 [120]. Since they occasionally precede the appearance of Lisch nodules, they can facilitate the diagnosis.

A unique, generally single, isolated and unilateral abnormality of a small second- or third-order **retinal venule**, which takes on a corkscrew-like tortuosity, can also be detected in a third of cases using direct ophthalmoscopy [121].

Congenital and acquired glaucoma, idiopathic congenital ptosis, and neurofibromas that impinge on the eyelid are other recognized complications of NF1 [119, 122].

### Optic pathway gliomas

Optic pathway gliomas (OPG) are benign tumors seen in 15 to 20% [123–128] of children with NF1. They usually appear early, in children younger than 6 years [128, 129], with a median age of clinical presentation at 4.2 years [123, 128]. Histologically these gliomas are juvenile pilocytic astrocytomas [129], they are slow growing with a low potential of malignancy. They frequently occur within the optic pathway including the optic nerve and optic chiasma [124, 130].

Their natural history is often indolent; however, due to their space occupying nature they can be locally invasive and become symptomatic in a good proportion of patients with NF1 [128, 131–134]. OPG can cause a rapid onset of proptosis associated with moderate-to-severe visual loss in the affected eye; or to abnormal ophthalmological examinations without any visual symptoms [123, 127, 129].. Precocious puberty can occur if the optic pathway tumor impinges on the optic chiasm [127, 135]. The risk of having a symptomatic optic glioma is greatest in children under 7 years; older patients rarely develop tumors that require medical intervention [129, 136].

Since infants and young children seldom complain of visual loss despite its severity, regular ophthalmological examinations are critical. All children diagnosed with NF1 should be subject to specific pediatric ophthalmological follow-up every year, at least up until the age of 13 years.

The exam should include measurement of visual acuity, confrontation visual field testing, color vision evaluation, and assessment of pupils, eyelids, ocular motility, irises, and fundi, with formal computerized or kinetic evaluation of visual fields as an adjunctive test if the patient is reliable, optical coherence tomography for quantification of retinal nerve fiber layer thickness. In case of equivocal results, visual evoked potential tests and/or imaging are indicated.

Finally, no specialized ophthalmological follow-up is necessary for adults with NF1 except for routine eye care.

Systematic imaging of the optic and cerebral pathways by MRI at the diagnosis of NF1 in young children without symptoms is controversial, and it should be requested only if an OPG is suspected [137]. There was no clear convincing benefit of systematic MRI screening in NF1 children under 6 years old as it had no influence on the therapeutic management of OPGs. Treatment of OPGs was initiated only when visual acuity was decreased (which can be detected clinically); and although MRI screening helped diagnosing OPGs earlier, treatment with chemotherapy did not improve the final visual outcome [138]. The main indication for neuroimaging should be determined by yearly clinical and ophthalmological assessments.

### Management of optic pathway gliomas

The natural history of OPG is variable and unpredictable in NF1 children, with absence of tumor progression in the majority of patients [127].

If a child with NF1 develops visual symptoms or physical signs (proptosis, decreased visual acuity, or precocious puberty) or an ophthalmological abnormality is detected, MRI of the brain and orbits to investigate for an OPG should be obtained. Long-term surveillance of OPGs is warranted, even though progression is rare after puberty.

Treatment is only necessary in the small percentage of patients who develop symptomatic tumors with clinically significant growth and progressive visual loss. The first-line treatment for most patients with symptomatic OPG is chemotherapy. Various chemotherapeutic agents have been used successfully and include carboplatin +/− vincristine, vinblastine, irinotecan and avastin [139–143]. They lead to radiological tumor regression; however, further randomized control trials are needed to compare them to one another. Unfortunately, very few children recover normal visual acuity after treatment [144].

Surgery has a very limited indication in the treatment of optic pathway gliomas as it can lead to permanent neurological damage [144] However, one can resort to surgery to remove large orbital tumors with no useful vision [144]. Surgical decompression of chiasmal gliomas is occasionally required especially in the context of third ventricular compression with secondary hydrocephalus [144].

Radiotherapy treatment of OPG is not recommended for children with NF1 due to the increased likelihood of the developing secondary malignancies, either gliomas or MPNST [98]; as well as developing neurovascular, endocrine and neuropsychological problems [144, 145].

#### Emerging evidence

Clinical trials assessing the use of mTOR and MEK inhibitors for the treatment of optic pathway gliomas are currently in progress (NCT01158651 and NCT02285439). Preliminary results of the NCT01089101 trial have shown that 10 of 25 (40%) with NF-associated OPG achieved partial response with selumetinib treatment (MEK1/2 inhibitor) and a progression-free survival of 96+/− 4%. Only one patient progressed while on treatment [146]. Bevacizumab (anti-VEGF antibody) alone or in combination with a chemotherapeutic agent has been shown to be a well tolerated and effective treatment for rapid tumor control to preserve vision and improve morbidity [147, 148].

- All children diagnosed with NF1 should be subject to specific pediatric ophthalmological follow-up every year, at least up until the age of 13 years.
- Lisch nodules are pigmented iris hamartomas and are found in all patients by the age of 30 years.
- Other ophthalmological manifestations of NF1 include: choroidal abnormalities, corkscrew-like tortuosity of retinal venule, congenital and acquired glaucoma, idiopathic congenital ptosis.
- Optic pathway gliomas (OPG) are benign tumors that usually appear in children younger than 6 years. Their natural history is often indolent; however, they can become symptomatic due to their space occupying nature. They can cause a rapid onset of proptosis associated with visual loss and precocious puberty.
- Systematic screening of the optic and cerebral pathways by MRI at the diagnosis of NF1 in young children without symptoms is not recommended. Neuroimaging should be determined by yearly clinical and ophthalmological assessments.
- Treatment is only necessary in the small percentage of patients who develop symptomatic tumors with clinically significant growth and progressive visual loss.
- The first-line treatment for most patients with symptomatic OPG is chemotherapy.

### Orthopedic manifestations

**Congenital dysplasia of the long bones** (mostly tibia but also fibula, radius and ulna) is a classic manifestation of NF1(7.2% )[149]. Bowing of long bones leads to visible deformity and fragile bone that is susceptible to fracture [150]. Repeated fractures with failure to heal can lead to the development of pseudarthrosis (failure of primary union of the separate bone ends can create a false joint) (2–3.6%) [149, 151]. The presence of bowing in an infant requires prompt radiographic assessment and referral to an orthopedic surgeon familiar with the management of NFl-related orthopedic problems in children.

The sphenoid bones comprise multiple ossification centers that fuse to become the important elements of the orbits. **Sphenoid wing dysplasia** is a distinctive feature of NF1 found in a minority of patients (1–7%) [15] and is often unilateral. Sometimes, absence or thinning of the sphenoid wing is secondary to the presence of an orbital plexiform neurofibroma [152]. In most cases, it is detected early in life and may progress over time. Patients with sphenoid wing dysplasia can also develop pulsating exophthalmus without visual loss; and absent sphenoid wing can lead to herniation of the temporal lobe into the orbit [122]. The treatment of sphenoid dysplasia is indicated in cases of pulsatile exophthalmia or in the context of surgery for plexiform NF. It should be carried out by a multidisciplinary team including cranio-facial teams familiar with this complex surgery.

**Scoliosis** is a common orthopedic manifestation in patients with NF1 (10–28%) [153] and often associated with vertebral dysplasia, which is found in more than 70% of NF1 patients on spinal MRI [154]. Therefore, patients with NF1 need yearly assessment of the spine during childhood and early adolescence. Patients with clinical evidence of scoliosis should have appropriate imaging and be referred to an orthopedist. Searching for dysplastic changes should be achieved meticulously in all patients since the management and prognosis of the scoliotic curve is essentially based on the presence of dystrophic features [155]. Scoliosis is generally classified into non-dystrophic and dystrophic types based on the absence or presence of skeletal dysplasia on plain radiographs. Non-dystrophic curvatures are usually found in adolescents with the same clinical and radiological features seen in idiopathic scoliosis and are managed similarly [156]. Dystrophic scoliosis is less common and often detected in early childhood. It is much more resistant to management and has a tendency for tremendous rapid progression with growth [6, 67]. Regular pulmonary function tests are indicated for patients with severe scoliosis. Dystrophic scoliosis usually requires early and aggressive corrective surgery with fusion the abnormal vertebral bodies.

#### Emerging evidence

A recent multicenter retrospective case series has confirmed that using growing rods is an effective fusionless method for controlling early-onset scoliosis associated with NF1 [157].

**Congenital thoracic deformities**, such as pectus excavatum or carinatum, have been reported in NF1 patients [158, 159].

Patients with NF1 are at higher risk of having **bone mineralization disorders** (osteopenia in 48% and osteoporosis in 25% of patients with NF1) [160, 161]. This is secondary to disorders of phosphorus and calcium metabolism, including vitamin D deficiency found in both children and adults [160]. Patients are at a high risk of bone fractures, due to congenital dysplasia and bone mineralization disorders [160].

It has been suggested that Vitamin D deficiency contributes to osteoporosis in NF1 [160, 162–164]. The impact of vitamin D supplementation in NF1 on bone density and fractures is unclear [160, 162]. In one retrospective study, vitamin D supplementation led to a significant reduction in the loss of bone-mineral density in adult NF1 patients whose vitamin D levels were maintained above 30 μg/L, compared with NF1 patients who had not been supplemented [165]. Further prospective studies are warranted to establish the need for vitamin D deficiency screening and appropriate replacements.

- Congenital dysplasia of the long bones is a classic manifestation of NF1. The presence of bowing in an infant requires prompt radiographic assessment and referral to an orthopedic surgeon familiar with the management of NFl-related orthopedic problems in children.
- Patients are at a high risk of bone fractures, due to congenital dysplasia and bone mineralization disorders (including phosphorus and calcium metabolism and vitamin D deficiency).
- Repeated fractures with failure to heal can lead to the development of pseudarthrosis
- Sphenoid wing dysplasia is a distinctive feature of NF1 found in a minority of patients detected early in life. It can be complicated by a pulsating exophthalmus or by herniation of the temporal lobe into the orbit. Surgery should be carried out by a multidisciplinary team familiar with this complex surgery.
- Scoliosis is common and often associated with vertebral dysplasia. NF1 patients need yearly assessment of the spine during childhood and early adolescence. Patients with clinical evidence of scoliosis should have appropriate imaging and be referred to an orthopedist. Scoliosis is generally classified into non-dystrophic and dystrophic types based on the absence or presence of skeletal dysplasia on plain radiographs.
- Regular pulmonary function tests are indicated for patients with severe scoliosis.
- Dystrophic scoliosis is less common, much more resistant to management and have a tendency for tremendous rapid progression with growth. Dystrophic scoliosis usually requires early and aggressive corrective surgery with fusion the abnormal vertebral bodies.
- Congenital thoracic deformities are found in 25% of patients.

### Endocrine manifestation

#### Puberty disorders and delayed growth

Short stature is found in a third of patients with NF1 and is not associated with disease severity [166]. Delayed puberty occurs in 20–30% of adolescents with NF1 [156]. On the other hand, precocious puberty is seen in 3% of patients [15]. Older children should be evaluated for early development of secondary sexual characteristics or abnormal growth acceleration as they may be related to an optic glioma involving the chiasm [127, 135].

#### Hormonal influence on neurofibromas

Various steroid hormone receptors have been found in neurofibromas tumor cells (receptors for estrogens, progesterone, growth hormone, and androgens) [167–169], potentially accounting for their growth and development at puberty or during pregnancy [170, 171]. Steroid hormones have been shown in vitro to initiate the growth of both neurofibromas and MPNSTs [172, 173]. However to date, there is not enough clinical or epidemiological data to contraindicate the use of these hormones in patients with NF1, including hormonal contraception.

- Patients with NF1 have a tendency for short stature and delayed puberty.
- Older children should be evaluated for abnormal growth acceleration or early development of secondary sexual characteristics as they may be related to an OPG impinging on the chiasm
- Although steroid hormones stimulate the growth of NFs and MPNSTs in vitro, there is not enough data to contraindicate the use of these hormones in NF 1 patients.

### Cardiovascular manifestations

**Hypertension** is a common finding in children with NF1 (16–19%) and increases with age [174–178]. Essential hypertension is the most common form in adults with NF1; however due to the lack of large studies, it is uncertain whether essential hypertension is a feature related to NF1 or just a coincidental disease.

Although essential hypertension is the most common cause of hypertension in NF1, it can also result from renovascular disease (such as renal artery stenosis), paragangliomas, pheochromocytoma, and coarctation of the aorta. Blood pressure should thus be evaluated at least annually in patients with NF1. Evaluation of renovascular causes should be initiated in patients with NF1 and hypertension [175] with appropriate imaging studies such as CT angiography of the renal arteries or arteriography. Laboratory evaluations should include serum creatinine and electrolytes, plasma renin, and urinalysis. These vascular malformations may recur after revascularization and long-term monitoring is therefore required [156].

Physicians should explore the presence of paragangliomas and pheochromocytoma in all NF1 patients with symptoms of catecholamine excess (sweating, palpitations, headache) and classically labile hypertension and/or hypertension refractory to standard treatment [156]. It typically presents in adult NF1 patients with a mean age at presentation of 40 years of age [179]. The diagnosis of a symptomatic pheochromocytoma or paraganglioma is based on the plasma and/or urinary free metanephrine levels and abdominal imaging. Any increase in metanephrine levels should be followed by imaging with MRI or CT scans examining the adrenal areas. F-DOPA PET may be useful for detecting extra-abdominal paragangliomas [180, 181]. Treatment involves alpha and beta blockade before surgery.

#### Emerging evidence

A recent prospective study on 234 patients with NF1 found the prevalence of pheochromocytoma to be 7.7%, which is well above that reported in previous studies [182]. They were asymptomatic in 80% of cases and non-secreting in 50% of cases. All non-secreting tumors were asymptomatic. The previous underestimated prevalence of pheochromocytomas in NF1 patients was attributed to the above described strategy whereby only symptomatic patients are subjected to screening. The authors of this study suggested that screening for pheochromocytoma should be undertaken in all NF1 patients starting at age 40 years, using an imaging modality at first, followed by a metaiodobenzylguanidine (MIBG) scan or a F-DOPA PET. Early detection of pheochromocytoma is important as it would allow for a tissue sparing surgery which is only possible when the tumor is smaller than 2 cm.

The incidence and type of **congenital heart defects** in individuals with NF1 were long undefined and not well-characterized. The previously reported frequencies of congenital heart defects ranged from 0.4 to 8.6% in 8 large series of NF1 patients [183–190]. However, the diagnosis of both NF1 and congenital heart disease was not clearly established in these patients and not distinguished from Watson and NF1-Noonan syndromes. Tedesco et al. were the first to evaluate the prevalence of cardiovascular abnormalities in patients with NF1 using echocardiography with color Doppler scan and found cardiac abnormalities in 13 of the 48 young patients (27%) [191]. The same group later described the cardiac abnormalities in 13 out of a total of 69 young patients (18.8%) with NF1 [192]. However, these studies are limited by their small sample size.

Vasculopathy is a common finding in NF1 patients and is a common cause of death in patients younger than 30 years of age [8]. It can affect any arterial vessel, leading to cerebrovascular events [193], renal artery stenosis [194], or peripheral vascular insufficiency [195]. One of the potentially severe manifestation of NF1 are cerebrovascular diseases which present as stenosis or occlusion of the internal carotid, middle cerebral and anterior cerebral arteries, moyamoya disease, and aneurysm formation [196, 197] .

NF1 patients have a propensity to bleed, in particular during surgery of neurofibromas [198]. Several reports have described hemorrhage into plexiform neurofibromas occurring spontaneously or after minimal trauma, as well as life-threatening bleeding during surgical excision [199, 200]. Bleeding has been attributed to both NF1-associated arterial dysplasia and primary hemostasis disorders [201]. Although studies have failed to find any hemostasis abnormalities in NF1 patients, careful assessment of hemostasis in NF1 patients may be warranted at least in those undergoing surgery [202–204].

- Hypertension is a common in patients with NF1 and blood pressure should be evaluated annually in patients with NF1.
- Essential hypertension is the most common cause of hypertension in NF1
- Hypertension can also result from renovascular disease, paragangliomas, pheochromocytoma, and coarctation of the aorta. Therefore, hypertensive NF1 patients should undergo a CT angiography of the renal arteries or arteriography. Laboratory evaluations should include serum creatinine and electrolytes, plasma renin, and urinalysis.
- Paragangliomas and pheochromocytoma are suspected in patients with symptoms of catecholamine excess (sweating, palpitations, headache), labile hypertension and/or hypertension refractory to standard treatment. The diagnosis is based on the plasma and/or urinary free metanephrine levels and abdominal imaging.
- Vasculopathy is a common finding in NF1 patients and is a common cause of death in patients younger than 30 years of age.

### Neurological evaluation

Neurofibromatosis type 1 can have an important impact on the central nervous system (CNS).

### Epilepsy

Compared to the general population, seizures are more common in individuals with NF1. It occurs in 8% of NF1 patients [205, 206] with an onset of epilepsy ranging from infancy to late middle age [207]. All seizure types are encountered but focal seizures are predominant [207]. Focal seizures may be due to an intracranial neoplasm [208]. Thus, the onset of seizures should lead to systematic neuroimaging searching for a lesion of the CNS (tumors, aqueduct stenosis, vasculopathy). Epilepsy is resistant to treatment in almost 30% of cases and the latter are associated with severe mental retardation [207].

### Cognitive impairment

The most common neurological complication is mild cognitive impairment [209]. Children with NF1 should have their developmental progress closely monitored with neurological and psychological screening evaluations early in life [210].

Neurocognitive impairment is a common manifestation of NF1, and includes an IQ in the low average range, behavioral problems and specific learning difficulties [5, 209, 211–214]. These learning difficulties include visuospatial and visuomotor impairments, language disorders, and fine and gross motor impairments, executive function problems. Attention-deficit hyperactivity disorder, behavioral abnormalities, autism spectrum disorders, and psychosocial problems can also be encountered in patients with NF1 [213, 215].

A delay in psychomotor and/or language development must prompt the physician to refer the child to the appropriate professional for early intervention and management. Attention-deficit disorder can be well managed with methylphenidate; and cognitive behavioral therapy can be helpful [209, 216].

#### Emerging evidence

Using a mouse model of NF1 with learning deficits, a preclinical trial established that it is an increased RAS/ERK signaling that is responsible for the deficits in neuronal plasticity along with the spatial and attention impairments. Subsequently, treatment with HMG-COA reductase inhibitors reversed these deficits in those mice [217]. Two randomized, controlled trials showed that simvastatin did not demonstrate an improvement in cognitive function [218, 219]. Although a phase-I study of lovastatin showed improvements in cognitive functioning [220], the phase-II trial did not reveal any benefit. So far there is not enough evidence to justify the use of statins in the treatment of cognitive impairment in NF1 individuals. An ongoing clinical trial is currently investigating the use of lamotrigine on cognition in NF1 (NCT02256124).

### Unidentified bright objects

Unidentified Bright Objects (UBO) are benign non-progressive lesions that appear as focal areas of high signal intensity on cerebral magnetic resonance T2-weighted images without a mass effect or contrast enhancement. They are most often found in the cerebellum, brainstem and basal ganglia of patients with NF1. They are more common in children than in adults with NF1 [221]. UBOs were found to be statistically associated with other NF1 manifestations such as brain tumors (including OPG), as well as language and spatial visualization problems [222–224].

They have been considered as useful diagnostic criteria for NF1 in young children. Indeed, several studies have shown a high sensitivity in children (ranging from 70 to 97%) and a high specificity (79–100%) for this diagnostic marker [225–227].

#### Emerging evidence

Over the past decade, new MRI based techniques have been introduced and have improved the sensitivity and specificity of detecting the UBOs even further [228–230].

- Epilepsy occurs in 8% of NF1 patients and focal seizures are the predominant type.
- The onset of seizures should lead to systematic neuroimaging searching for a lesion of the CNS (tumors, aqueduct stenosis, vasculopathy).
- Epilepsy resistant to treatment is associated with severe mental retardation
- The most common neurological complication is mild cognitive impairment.
- Children with NF1 should have their developmental progress closely monitored with neurological and psychological screening evaluations early in life.
- Neurocognitive impairment includes an IQ in the low average range, behavioral problems and specific learning difficulties.
- A delay in psychomotor and/or language development must prompt the physician to refer the child to the appropriate professional for early intervention and management.
- UBO are benign non-progressive lesions without a mass effect that have been considered as useful diagnostic criteria for NF1 in young children with high sensitivity and specificity.

### Oncological manifestations

NF1 is associated with an increased risk of malignancy and a life expectancy about 10–15 years shorter than the general population. Malignancies are the leading cause of death in NF1 [10, 231]. A patient with NF1 is four times more likely to develop a malignancy as compared to the general population [13, 232–234].

As compared to the general population, NF1 patients are 2–3 times more likely to develop a cancer of the esophagus, stomach, colon and lung; 3–7 times more likely to develop a cancer of the liver, thyroid, ovary, breast, malignant melanoma, non-Hodgkin’s lymphoma and chronic myeloid leukemia; 15 times more likely to develop small intestine tumors and 20 times more likely to develop bone cancer [232].

Patients with NF1 should follow the same screening guidelines as those for the general population [156].

The field of breast cancer in patients with NF1 has seen increasing attention in the past few years. Indeed, several studies have demonstrated that breast cancer in NF1 patients affects primarily women younger than age 50 years [11, 13, 235–237]; with mortality rates higher than those for women with breast cancer in the general population [14]. Based on this increased risk of early-onset breast cancer in female patients with NF1, annual breast screening with mammography was recommended by expert opinion to begin at age 40 years [236]. Treatment of NF1-associated breast cancer is similar to that of breast cancer in the general population.

#### Emerging evidence

A population-based study in Finland of 1404 NF1 patients showed a significant increased risk of breast cancer in NF1 patients (standardized incidence ratio (SIR) 3.04; 95% CI, 2.06–4.31; *P* < .001) [237], with the highest incidence in NF1 women younger than 40 years of age. Furthermore, NF1-associated breast cancer was associated with poorer survival compared to breast cancer among the general population [237]. While all studies confirm the higher incidence of breast cancer in NF1 women younger than 50 years of age; studies diverge when it comes to the increased risk of breast cancer in NF1 women above the age of 50. The finish stud y[237] and a retrospective review (*n* = 76) in the United States [235] respectively showed a two-fold and 2.8-fold increased risk of breast cancer in patients with NF1 over age 50 years. On the other hand, a prospective study of NF1 patients from the United Kingdom (*n* = 227) and a retrospective review in the United States (*n* = 126) showed that breast cancer risk in NF1 patients is not significantly increased beyond the age of 50 years [11, 13].

Given the increased risk of early-onset breast cancer in patient with NF1, the most recent version of the North American National Comprehensive Cancer Network (NCCN guidelines) (Genetic/Familial High-Risk Assessment: Breast and Ovarian, Version 2.2017) advise annual mammogram starting at age 30 and consideration of breast MRI with contrast from ages 30 to 50 in the NF1 population [238]. These NCCN guidelines suggested that using breast MRI as a screening tool in patients with NF1 may be discontinued starting the age of 50 years on the basis that breast cancer risk in NF1 patients above 50 years of age did not significantly differ from that of women in the general population in the above-mentioned studies [13, 238]. However, large prospective studies are needed in order to construct formal recommendations for this special population, including screening above the age of 50. In light of the most recent evidence, the upcoming updated version of the PNDS, will decrease the age at which to begin breast cancer screening with imaging to 30 years of age.

The presence of multiple cutaneous neurofibromas makes both self-breast examination and physical examination difficult for NF-1 patients and may obscure a small breast lump; screening should therefore rely on breast mammography or MRI. However, female patients should be encouraged to regularly examine their breasts; and the discovery of a new lump should directly prompt imaging.

Although digital mammography is the gold standard for screening for early stage breast cancer, interpreting images of a large breast carcinoma in an NF1 patient is challenging due to the high number of neurofibromas and screening with breast MRI should be considered. However, early screening generates two major concerns: First, the safety of mammography in NF1 patients, especially if started at a very young age, is unknown. Although the radiation exposure is low with mammography, NF1 patients have been shown to develop secondary malignancies in response to therapeutic ionizing radiation [98]. Second, the lower specificity of MRI may lead to overdiagnosis with the unnecessary core biopsy of lesions that may turn out to be benign neurofibroma rather than breast cancer [239]. Risk-reducing mastectomy is not recommended in NF1 patients as there are no data regarding its benefit; however, it may be suggested based on family history.

##### Central nervous system tumors

The most common brain tumor affecting individuals with NF1 is the OPG seen in 15 to 20% of children with NF1 [123–128]. OPGs are discussed extensively in the Ophthalmological manifestations section.

#### Brainstem Gliomas (BSG)

The second most frequently encountered brain tumor in individuals with NF1 is the BSG [240]. These are indolent tumors that arise in slightly older children and are often discovered incidentally on neuroimaging studies [241–243]. Similar to OPGs, these tumors are usually pilocytic astrocytomas. They might come to medical attention with headache, nausea, vomiting, cranial neuropathies, and ataxia [241]. Observation is recommended for asymptomatic children. These tumors might cause obstructive hydrocephalus requiring ventriculoperitoneal shunt placement or an endoscopic ventriculostomy [241]. Treatment with carboplatin and vincristine chemotherapy is reserved for those with progressive or worsening symptoms [139].

##### Gastrointestinal neuroendocrine tumors

Gastrointestinal stromal tumors (GISTs) are soft-tissue sarcomas that can be located in any part of the digestive system. In patients with NF1, GISTs tend to occur at an earlier age, are often multiple and frequently occur in the small intestine [244, 245]. The most common symptoms reported are abdominal pain, intestinal obstruction, bleeding and intestinal perforation [246, 247]. These tumors do not harbor the mutations in *KIT* and *PDGFRA*, which are typically associated with sporadic GISTs. These tumors are therefore poorly responsive to the tyrosine kinase inhibitor imatinib [244, 248, 249], although sunitinib, another tyrosine kinase receptor inhibitor, can be useful in metastatic disease [250, 251]. The treatment of NF1-associated GIST is complete surgical resection.

Rare neuroendocrine tumors that originate from endocrine cells within the gastrointestinal tract (duodenal somatostatinoma, pancreatic somatostatinoma and insulinoma, carcinoid tumors of the small intestine) and have also been reported in patients with NF1 [252]. Their diagnosis should prompt a search for Multiple Endocrine Neoplasia type1 [253].

- NF1 is associated with an increased risk of malignancy and malignancies are the leading cause of death in NF1.
- Given this higher risk, thorough clinical examination should be performed regularly, at each visit, with referral to appropriate specialists and oncologists when needed.
- Breast cancer in NF1 patients affects primarily women younger than 50 years, although some studies suggest an increased risk in older patients as well.
- Patients with NF1 should follow the same screening guidelines as those for the general population. However, women with NF1 should undergo regular mammography-based screening starting the age of 40 years. In light of the most recent evidence, the upcoming updated version of the PNDS, will decrease the age at which to begin breast cancer screening with imaging to 30 years of age.
- The most common brain tumor affecting individuals with NF1 is the OPG seen in 15 to 20% of children with NF1
- In patients with NF1, GISTs tend to occur at an earlier age, are often multiple and frequently occur in the small intestine. These tumors do not harbor the mutations in *KIT* and *PDGFRA*. They are therefore poorly responsive to the tyrosine kinase inhibitor imatinib. The treatment of NF1-associated GIST is complete surgical resection.

## Follow up and management of specific cases

The medical follow up of patients with NF1 relies on active partnership between multiple health care providers using a multidisciplinary approach.

Lifetime monitoring is recommended as soon as the diagnosis of NF1 is suspected. Clinical evaluation by a NF1 specialist should take place on a yearly basis for both children and adults with a high-risk phenotype. Otherwise, NF1 patients without the high-risk phenotype or complications should visit the NF1 specialist every two to 3 years, with the rest of the visits taking place annually with a primary care physician, dermatologist, or pediatrician [156].

Annual clinical examinations allow for early detection of complications, decreasing morbidity and improving quality of life. Routine screening investigations are not recommended, and their request should be guided by a thorough clinical evaluation. This monitoring should be carried out within the framework of multidisciplinary management, in collaboration with the patient’s general practitioner. Children and adults with high-risk phenotype should be followed up by a specialized NF1 team.

A complete clinical examination, including blood pressure measurement, should be carried out at each consultation. Annual examinations allow for early detection of complications, including the physical and psychological impacts of the disease on patients. This in turn improves the management of NF1 patients with proper timely referral to specialist teams.

Table 2 summarizes the screening modalities to be undertaken in the medical follow up of patients with NF1.

**Table 2:** Screening for major NF1 complications

Sought Complications		Affected patients	Screening modality
Dermatological manifestations	Subcutaneous, internal, and plexiform NF: malignant transformation? Esthetic or functional problems?	Children, adults	Clinical examination: Pain, neurological deficit, increase in size, functional and psychological repercussions Additional examinations: optional Indications: suspicion of malignancy, preoperative, internal NF risk factor
	Juvenile xanthogranuloma (JXG)	Children	Physical examination If JXG present: palpation of ganglionic areas and complete blood count
Orthopedic manifestations	Bone dysplasia and pseudarthrosis of the long bones, fractures	Children, adults	Clinical examination: search for gibbosity, bone deformity. X-ray if abnormalities found on clinical examination
	Scoliosis	Children, adults	Physical examination Additional examinations (optional): Front and profile X-ray views of the spine if clinical abnormalities found (1st line) MRI should be reserved for forms with vertebral and/or costal dysplasia (expert consensus) Pulmonary function tests to evaluate the impact of severe scoliosis
	Bone mineralization disorder, osteoporosis	Children, adults	Consider bone densitometry scans based on clinical examination, vitamin D levels and X-ray results
Endocrinological manifestations	Pubertal and growth disorders	Children	Follow pubertal development and the growth curve, measure head circumference.
Cardiac and vascular manifestations	Essential and secondary hypertension	Children, adults	Physical examination: Blood pressure measurement at each consultation (at least annually), discuss the possibility of ambulatory measurement Look for signs suggestive of pheochromocytoma Additional examinations if high blood pressure. As a first-line examination: angio-CT scan of the renal arteries and abdominal CT Plasma and/or urinary determination of metanephrines in adults.
	Cardiac abnormalities	Children, adults	Clinical examination
	Hemorrhagic manifestations	Children, adults	Assess hemostasis before any surgical, dental or obstetric procedure.
Pain, psychological repercussions, quality of life		Children, adults	Clinical examination Offer psychological counseling, pain specialist referral
Otolaryngologic manifestations	Deafness, neurinoma, phonatory disorder, laryngeal NF	Children, adults	Otolaryngologic examination with tuning fork
Neurological manifestations	OPG	Children	Interview: repeated falls leading to suspicion of decrease visual acuity or visual field amputation Neurological and ocular examination: strabismus, nystagmus, low visual acuity, neurological deficit, signs of intracranial hypertension. Early puberty, deviation from the growth curve, measurement of head circumference Ophthalmological screening at least once per year until the age of 13 years and then if signs appear MRI of the optic and cerebral pathways is not systematic and should be done only if suspicion of OPG
	Epilepsy, hydrocephalus, intracranial hypertension, stroke, headache	Children, adults	Neurological examination Cerebral MRI and electroencephalogram guided by the abnormalities detected on clinical examination
	Developmental delay, learning difficulties, behavioral problems	Children	Evaluation of psychomotor development and academic proficiency at each consultation Search for learning difficulties Comprehensive neuropsychomotor assessment before entering elementary school, support for school integration
	Medullary and nerve compression, peripheral neuropathy, Socio-professional integration	Adults	Clinical examination
Cancers	MPNST (60% of cancers in NF1 patients)	Children, adults	Clinical examination: recent increase in size of plexiform NF, appearance of pain. Additional examinations if signs appear If high NF-1 score: screening for internal neurofibromas by imaging (preferably by MRI).
	All other cancers	Children, adults	Clinical examination: asthenia, high blood pressure, intracranial hypertension symptoms, abdominal mass, bladder signs, appearance of mass, compressive syndrome … Screening identical to that of the general population except for earlier breast screening (> 40 years)

### Defining a “high-risk” subpopulation

MPNSTs are among the main causes of death in adults with NF-1 [88]. The major risk factor for the development of MPNST is the presence of many subcutaneous neurofibromas, often associated with peripheral neuropathy and the presence of at least one internal neurofibromas [56, 96]. In order to optimize the surveillance of patients with NF1, a “high-risk” subpopulation that is most likely to develop MPNST and to require close monitoring was defined.

There exists a very strong association between the presence of internal neurofibromas and MPNSTs [57]. Whole-body MRI of NF1 patients allows assessment of the burden of internal neurofibromas. However, screening MRI for the detection of an internal neurofibroma or MPNST is not systematically recommended in patients with NF1.

Since MPNSTs develop from internal neurofibromas, a clinical score (NF-1 score) for predicting internal neurofibromas in adults (age > 17 years) was developed and validated (Table 3) to help orient physicians towards imaging studies [58]. Four variables were independently associated with internal neurofibromas: at least two subcutaneous neurofibromas, age ≤ 30, absence of cutaneous neurofibromas, and fewer than six café-au-lait macules [58]. This might improve the early diagnosis of MPNSTs, as close monitoring could be offered to patients with high-risk score values. A high NF-1 score warrants screening for internal neurofibromas by imaging (preferably by MRI) [156]. If no internal neurofibromas are detected, then there is no need to repeat the imaging. If, on the other hand, internal neurofibromas are present, they should be monitored clinically with new imaging undertaken only when symptoms appear.

**Table 3:** NF1 score clinical score for predicting internal neurofibromas in adults

NF1 score	
Independent factors associated with the presence of internal NFs	Points
Age ≤ 30 years	10
Absence of cutaneous NFs	10
≥ 2 subcutaneous NFs	15
< 6 café-au-lait macules	5
Probabilities of the presence of internal neurofibromas according to the NF-1Score	
NF1- Score	Probability (%)
0	5.1
5	8.3%
10	13.3%
15	20.7%
20	30.8%
25	43%
30	56.1%
35	68.4%
40	78.7%

### Pain evaluation

Pain is found in around 7% of patients and is a very common reason for consultation [156]. The classical manifestations of NF1 such as nodular neurofibromas (subcutaneous or internal), plexiform neurofibromas and skeletal deformations can all lead to chronic pain. At each visit, patients should be asked, specifically about any change in pain associated with a preexisting plexiform neurofibroma to rule out the possibility of a malignant transformation into a MPNST.

Pain control in patients with NF1 follows the general pain management guidelines; whereby the prescribed drug is chosen from the “analgesic ladder” depending on pain severity. Antidepressants or antiepileptic agents are the preferred treatments for neuropathic pain. Complementary nonpharmacological therapy, such as physiotherapy, occupational therapy and functional rehabilitation should also be presented to patients.

### Quality of life evaluation

The quality of life (QOL) in patients with NF1 is often reduced, even in patients with a mild phenotype or a very small affected area [254–256]. Disease acceptance and self-esteem preservation vary considerably between individuals. Physicians should therefore not make assumptions about the psychological impact of NF1 on a particular patient and QOL evaluation must therefore be systematic and regularly repeated.

#### Emerging evidence

The impact of NF1 on Quality of Life (INF1-QOL) questionnaire is a reliable, recently validated disease specific questionnaire that correlates moderately well with disease severity as it comprises a broad scope of themes related to NF1 manifestations. It has the major advantage of being quick and simple to complete [257].

### Physical impact evaluation

Evaluation the physical impact of the disease on patients with NF1 is important as it improves their management and early referral to specialist teams. This assessment can be achieved using scales that are not specific for NF1.

The Riccardi’s severity index is used to evaluate the severity of NF1 based on the extent of cutaneous involvement and disabling complications [258, 259]. Ablon’s visibility index can be used to grade the cosmetic disfigurement of NF1 [260].

Nevertheless, the doctor-patient relationship remains fundamental for the assessment of the physical and psychological impact. Self-assessment scales such as the Skindex [261], SF-36 (Short Form 36 health survey) [262, 263], Children’s Dermatology Life Quality Index (CDLQI) and DISABKIDS [156] are other useful tools.

### Psychological support

Psychological support can be offered to patients and their families at diagnosis, while delivering bad news regarding disease complications or heavy surgical interventions, as well as during pregnancy planning.

The esthetic complications of the disease, the chronic pain, the cognitive impairment and learning disabilities, pregnancy planning, and the complex and unpredictable nature of NF1 can all contribute to psychological distress (anxiety, decreased self-esteem, social isolation) with a negative impact on relationships and functionality. Since patients are often reluctant to raise these issues, the clinician should evaluate systematically the need for psychological support for patients and their families and offer it when needed.

### Social support

Social support should be offered to patients and their families, along with guidance and advise through the various domains of social intervention: handicap, obtaining social security rights, workforce integration, home support, school placement, among others. The social support should factor in the medical, psychological and environmental impacts on individuals with NF1, to ensure optimal outcome.

- Lifetime monitoring of NF 1 patient is recommended.
- Children and adults with a high-risk phenotype should be evaluated clinically by a NF1 specialist on a yearly basis.
- A “high-risk” subpopulation that is most likely to develop MPNST was defined. The major risk factor is the presence of many subcutaneous neurofibromas, often associated with peripheral neuropathy and the presence of at least one internal neurofibromas [56, 96].
- A validated NF-1 score clinical score for predicting internal neurofibromas in adults was developed. A high NF-1 score warrants screening for internal neurofibromas by imaging (preferably by MRI).
- At each visit, patients should be asked, specifically about any change in pain associated with a preexisting plexiform neurofibroma to rule out the possibility of a malignant transformation into a MPNST.
- Pain control in patients with NF1 follows the general pain management guidelines. Antidepressants or antiepileptic agents are the preferred treatments for neuropathic pain.
- The quality of life in patients with NF1 is often reduced, even in patients with a mild phenotype or a very small affected area. QOL evaluation must be systematic and regularly repeated.
- Various scales are available for the evaluation the physical impact of the disease on patients with NF1.
- The need for psychological support for patients and their families should be evaluated systematically and offer it when needed.
- Social support should be offered to patients and their families, along with guidance and advise through the various domains of social intervention.

### Pregnancy

Pregnancy is not contraindicated in female patients with NF1; however, careful evaluation with close follow up of their pregnancies is warranted. Hormonal changes associated with pregnancy might cause the appearance of new neurofibromas and an increase in the size of existing neurofibromas [264]. Although maternal mortality does not appear to be increased, pregnant women with NF1 may have increased morbidity, particularly hypertension, preeclampsia, placental abruptions, and vascular complications [265, 266]. Cesarean deliveries are more common among NF1 patients [264, 266–268]. Fetal complications include preterm birth and intra uterine growth restriction [267, 268].

### Segmental NF

Segmental neurofibromatosis is a rare variant of NF1 (estimated prevalence between 0.0014 and 0.002%) characterized by neurofibromas and/or café-au-lait macules localized to one body segment with no crossing of the midline and no family history (since it results from a post-zygotic *NF1* mutation leading to somatic mosaicism) [269]. It is typically unilateral but can also be bilateral in 6% of cases, either in a symmetric or asymmetrical distribution [27].

Malignant transformations of plexiform NF into MPNST has been reported warranting a regular clinical monitoring of these patients [270]. The prognosis of patients with segmental neurofibromatosis is better than that of NF1 patients, nevertheless one study suggested a possible increased risk of certain malignancies [271].

The risk of having a child with NF1 is roughly 5% for a parent with segmental disease [27] and hence genetic counselling should be offered to these patients.

## Conclusion

In summary, this PNDS can be used by healthcare providers as guidance for the management of NF1 patient as it provides an in-depth follow-up strategy of NF1 patients. Lifetime monitoring begins as soon as the diagnosis of NF1 is suspected. Given the complexity of the disease, the management of children and adults with NF1 entails the implication of the full complement healthcare providers and communication among the various specialties. Further studies are emerging and will hopefully help further elaborate optimal strategies of disease management. Lastly, evolving understandings of the molecular pathogenesis of NF1 and the elaboration of specific preclinical mice models of NF1-associated malignant disease provide promising grounds for the conception of innovative rational molecular-targeted drugs [272].

## Data Availability

Data sharing not applicable to this article as no datasets were generated or analysed during the current study.

## Abbreviations

CALMs: Café-au-lait macules
CNS: Central Nervous System
CT: Computed tomography
FDG: [18F]2-fluoro-2-dexoy-D-glucose
JMML: Juvenile myelomonocytic leukemia
JXG: Juvenile xanthogranulomas
MPNST: Malignant peripheral nerve sheath tumors
MRI: Magnetic resonance imaging
NF: Neurofibromas
NF1: Neurofibromatosis type 1
NIH: National Institutes of Health
OPG: Optic pathway gliomas
PET: Positron emission tomography
PNDS: Protocole national de diagnostic et de soins
QOL: Quality of life
UBO: Unidentified Bright Objects
